# Characterization of virulence genes in *Escherichia coli* strains isolated from pre-weaned calves in the Republic of Korea

**DOI:** 10.1186/s13028-020-00543-1

**Published:** 2020-08-20

**Authors:** Ji-Hyoung Ryu, SuHee Kim, Jinho Park, Kyoung-Seong Choi

**Affiliations:** 1 Department of Animal Science and Biotechnology, College of Ecology and Environmental Science, Kyungpook National University, Sangju, 37224 Republic of Korea; 2 Gyeongsang National University Hospital, Jinju, 52727 Republic of Korea; 3 College of Veterinary Medicine, Jeonbuk National University, Iksan, 54596 Republic of Korea; 4 Present Address: Foreign Animal Disease Division, Animal and Plant Quarantine Agency, Gimcheon, 39660 Republic of Korea

**Keywords:** Diarrhea, ETEC, *F17*, STEC, Pre-weaned calves, Virulence genes

## Abstract

**Background:**

*Escherichia coli* is an important cause of diarrhea in calves and its diarrheagenic properties are related to presence of certain virulence genes. In this study, the prevalence of virulence genes *F5*, *F17*, *F41*, *sta*, *stx1*, *stx2*, *eae*, and *saa* in *E. coli* isolated from pre-weaned calves presenting with (n= 329) or without diarrhea (n= 360) was explored using multiplex polymerase chain reaction. We also evaluated the association between detection of *E. coli* and the presence of diarrhea.

**Results:**

*Escherichia coli* was detected in 56.3% (388/689) of the fecal samples and showed the highest prevalence (66.5%) in 21–40-day-old calves and the lowest (46.3%) among those that were 1–20 days old. The prevalence of the enterotoxigenic *E. coli* (ETEC) and Shiga toxin-producing *E. coli* (STEC) pathotypes was detected in 73.9% and 15.9%, respectively. The results showed no association between diarrhea and the presence of *E. coli* in general, ETEC or STEC. The *F17* gene was the most frequently detected virulence factor in *E. coli* of calves of all ages regardless of diarrhea. Interestingly, the results show that the calves aged 41–60 days with *F17*-positive *E. coli* are at a higher risk for production of Shiga toxin (*Stx1*; 95% confidence intervals: 1.86–31.95; P = 0.005) compared to calves aged 1–20 days; no association between this finding and diarrhea was observed among the calves of this age group. Moreover, the virulence genes associated with the ETEC and STEC strains were not significantly associated with pathogenicity in this study cohort.

**Conclusions:**

These results suggest that while the incidence of *E. coli* is age-related, there was no relationship linking *E. coli* virulence genes to calf age and diarrhea. Furthermore, the present study demonstrated that detection of *E. coli* strains either with or without virulence factors was not associated with diarrhea in pre-weaned calves.

## Background

*Escherichia coli* is an important cause of diarrhea in calves and has negative economic effects on the livestock industry worldwide due to high mortality and reduced growth rate [1]. Diarrheagenic *E. coli* are divided into six pathotypes based on the virulence properties of the bacteria and resulting clinical signs of the host; these include enterotoxigenic *E. coli* (ETEC), enteropathogenic *E. coli* (EPEC), Shiga toxin-producing *E. coli* (STEC; e.g., enterohemorrhagic *E. coli* [EHEC]), enteroinvasive *E. coli* (EIEC), enteroaggregative *E. coli* (EAEC), and diffusely adherent *E. coli* (DAEC) [2].

Of the six pathotypes, ETEC has been identified as a the major causative agent of calf diarrhea, whereas the others are frequently isolated from diarrheic as well as normal feces [3]. The main virulence factors contributing to diarrhea-associated ETEC are fimbrial adhesins and enterotoxins. Fimbrial adhesins (F5, F17, and F41) mediate the attachment of bacteria to epithelial surfaces in the small intestines, thereby facilitating bacterial colonization with both heat-labile (LT) and heat-stable (STa and STb) enterotoxins; the toxins promote secretion of fluids and electrolytes from the intestinal epithelial cells, which ultimately results in diarrhea [4–6]. The role of the STEC as an etiological agent of calf diarrhea is not yet conclusive; however, there are reports of cases of fatal STEC infection in cattle [3]. In humans, infection with STEC can result in hemorrhagic colitis, and hemolytic uremic syndrome (HUS) [7, 8]. STEC produces the Shiga toxins, Shiga toxin 1 (Stx1) and Shiga toxin 2 (Stx2), and expresses several virulence factors, including intimin (encoded by the *eae* gene) [9] and the STEC auto-agglutinating adhesin (*saa*) [10]. Domestic ruminants, particularly cattle, are known as the most important reservoirs of STEC.

The fimbrial adhesins, F17, F5, and F41 are mainly associated with ETEC in calves [11]. Likewise, the F5 and F41 virulence factors have been associated with diarrhea in animals [3, 12], although these genes are detected at decreased frequency with increasing age. *E. coli*-associated F17 fimbrial adhesions have been identified from animals and from humans with diarrhea and/or septicemia [13]. By contrast, STEC is not typically associated with disease in cattle and is detected more frequently in healthy animals [3]. However, STEC carried by healthy cattle may pose a potential risk to humans. The objective of the present study was to investigate the role of *E. coli* as a causative agent of diarrhea, to explore the prevalence of virulence genes associated with ETEC (*F5*, *F17*, *F41*, and *sta*) and with STEC (*stx1*, *stx2*, *eae*, and *saa*), and to determine the relationship between virulence genes and diarrhea in pre-weaned calves in the Republic of Korea (ROK).

## Methods

### Fecal samples

Fecal samples were collected from a total of 689 pre-weaned calves that were less than 61 days old during March 2017 and October 2018. The samples were taken from nine different regions within the ROK, including Anseong (*n *= 39), Asan (*n *= 95), Geochang (*n *= 37), Gimje (*n *= 106), Gyeongju (*n *= 6), Mungyeong (*n *= 112), Samrye (*n *= 80), Sangju (*n *= 8), and Yeongju (*n *= 206). These herds were maintained as part of a large-scale cattle breeding system; the calves had access to water and concentrates. All calves were fed with colostrum from birth. All fecal samples were collected directly from the calves by rectal palpation and were stored in plastic containers. Fecal samples were categorized based on their consistency using the scoring system (0 to 3) included in the calf health scoring guide created by the University of Wisconsin-Madison School of Veterinary Medicine (https://fyi.extension.wisc.edu/heifermgmt/files/2015/02/calf_health_scoring_chart.pdf). All samples scored at 2 or 3 were categorized as diarrhea. The fecal samples were classified as normal (*n *= 360) or diarrheic, typically with a pasty and watery (*n *= 329) consistency. Most calves used in this study were able to stand and suck at feeding time but preferred to remain in sternal recumbency. Of the 689 samples obtained, 140, 197, 129, 89, 79, and 55 were from calves at 1–10 days, 11–20 days, 21–30 days, 31–40 days, 41–50 days, and 51–60 days of age, respectively. The collected fecal samples were placed in 50 mL tubes and shipped on ice to the Animal Immunology Laboratory at Kyungpook National University.

### *E. coli* culture, DNA extraction, and PCR

Each fecal sample was inoculated onto MacConkey agar (MBcell, Seoul, ROK) and blood agar (Asan Pharmaceutical Co., Ltd., ROK) and was incubated for 12–18 h at 37 °C. Pure colonies with appearance suggestive of *E. coli* were selected at random; identities were confirmed via standard biochemical tests (API 20E System, bioMérieux, France). From each sample, 2 to 10 colonies were selected, suspended in 200 μL of water, and boiled for 10 min. After centrifugation at 12,000×*g* for 10 min, the supernatant was used as a template for polymerase chain reaction (PCR). PCR for ETEC and STEC was performed by two multiplex PCR strategies; the first reaction targeted two factors (*F17* and *saa*) and the second targeted six factors (*F*5 (= *K99*), *F41*, *stx*1, *stx*2, *eae*, and *sta*) as previously described [14–17]. PCR cycling conditions for *F17* and *saa* included 95 °C for 10 min followed by 30 cycles of 30 s at 95 °C, annealing at 61 °C for 30 s, and 72 °C for 30 s. PCR cycling conditions for *eae*, *F5*, *F41*, *stx1*, *stx*2, and *sta* included 95 °C for 10 min, followed by 25 cycles of denaturing at 95 °C for 30 s, annealing at 50 °C for 45 s, and at 70 °C for 90 s. The reaction mixtures contained 2 μL of DNA template, 10 pM of each primer, and 10 μL of EmeraldAmp^®^ GT PCR Master Mix (Takara, Shiga, Kusatsu, Japan) in a final volume of 20 μL. The positive controls for each target gene were obtained from Animal and Plant Quarantine Agency (Gimcheon, ROK), and distilled water was used as a negative (no template) control in each PCR experiment. Reactions were conducted in an automated DNA thermal cycler (Bio-Rad, Hercules, CA, USA). PCR products were visualized under UV light after 1.5% agarose gel electrophoresis and ethidium bromide staining.

### Statistical analysis

Statistical analysis was performed using the SPSS Statistics 25 software package for Windows (SPSS Inc, Chicago, IL, USA). Binary and multinomial logistic regression analysis was used to evaluate the prevalence of *E. coli* and to determine any associations between the prevalence and specific independent variables, including age, virulence genes, and fecal consistency. Odd ratios (OR) with 95% confidence intervals (CIs) were calculated to assess the association between age and prevalence of *E. coli* and virulence genes. A P-value of less than 0.05 was considered to be statistically significant.

## Results

### Prevalence of *E. coli* according to age in pre-weaned calves

*Escherichia coli* was detected in 388 of a total 689 (56.3%) fecal samples. *E. coli* was found with a prevalence rate of 58.3% in normal feces and 54.1% in diarrheic feces, respectively. These results indicated that *E. coli* was not directly associated with diarrhea in pre-weaned calves (Table 1, P = 0.420). The age distribution relative to detection of *E. coli* is shown in Table 1. The prevalence of *E. coli* was significantly associated with age (P < 0.001). *E. coli* was detected in 66.5% of the samples from calves aged 21–40 days compared to only 46.3% of those from the 1–20-day-old group. The binomial regression analysis revealed that the prevalence of *E. coli* increased 2.31-fold in calves aged 21–40 days (95% CI 1.62–3.28; P < 0.001), and 2.15-fold in those aged 41–60 days (95% CI 1.42–3.25; P < 0.001) compared to that observed in calves aged 1–20 days (Table 2).

**Table 1:** Prevalence of *Escherichia coli* based on fecal consistency and age in pre-weaned calves (n= 689) in the Republic of Korea

Variables	No. (%) of *E. coli*-positive samples	No. (%) of *E. coli*-negative samples	Total	χ^2^ (P value)
Fecal consistency				
Diarrheic	178 (54.1%)	151 (45.9%)	329	0.651 (0.420)
Normal	210 (58.3%)	150 (41.7%)	360	
Age (days)				
1–20	156 (46.3%)	181 (53.7%)	337	26.205 (< 0.001)
21–40	145 (66.5%)	73 (33.5%)	218	
41–60	87 (64.9%)	47 (35.1%)	134	
Fecal consistency × age				4.746 (0.093)

**Table 2:** Univariate binomial logistic regression analysis for age distribution and detection of *Escherichia coli* in fecal samples

Age (days)	*E. coli*		
	OR	P value	95% CI
1–20 (ref.)	1.00	–	–
21–40	2.31	< 0.001	1.62–3.28
41–60	2.15	< 0.001	1.42–3.25

### Distribution of virulence factors

The distribution of virulence factors characteristic of ETEC (*F5*, *F17*, *F41*, and *sta*) and STEC (*stx1*, *stx2*, *eae*, and *saa*) was compared to fecal consistency groups. In this study, ETEC-associated virulence factors were identified in fecal samples from 287 calves (73.9%) and STEC-associated virulence factors in samples from 61 calves (15.7%). Of all the virulence factors for ETEC and STEC evaluated, the *F17* gene showed the highest prevalence (72.2%), followed by the *stx1* gene (9.3%), *eae* gene (4.4%), and *saa* gene (1.8%) regardless of diarrhea (Table 3). Combinations of two or three virulence genes were also observed in both calves with and without diarrhea. *Stx1 *+ *eae*, *F17 *+ *stx1*, and *F17 *+ *stx1 *+ *eae* were detected in 3.6%, 3.1%, and 0.5% of the fecal samples, respectively (Table 3). There were no associations between diarrhea and any pattern of virulence genes evaluated in this study.

**Table 3:** Association between diarrhea and virulence genes (single or in combination) in fecal samples from pre-weaned calves

Virulence genes	No. (%) of *E. coli* strains by the presence of diarrhea		P value
	Diarrheic calves (n= 329)	Healthy calves (n= 360)	
*F17*	124 (37.7)	156 (43.3)	0.246
*F5*	2 (0.6)	1 (0.3)	0.600
*F41*	0 (0.0)	0 (0.0)	–
*sta*	1 (0.3)	1 (0.3)	0.935
*stx1*	17 (5.2)	19 (5.3)	0.682
*stx2*	0 (0)	1 (0.3)	0.997
*eae*	8 (2.4)	9 (2.5)	0.951
*saa*	5 (1.5)	2 (0.6)	0.322
*F17 *+ *F5*	2 (0.6)	1 (0.3)	0.607
*F17 *+ *stx1*	5 (1.5)	7 (1.9)	0.529
*F17 *+ *F41*	2 (0.6)	1 (0.3)	0.449
*F17 *+ *eae*	2 (0.6)	1 (0.3)	0.463
*stx1 *+ *eae*	6 (1.8)	8 (2.2)	0.600
*stx1 *+ *saa*	1 (0.3)	1 (0.3)	0.825
*stx1 *+ *F5*	0 (0.0)	1 (0.3)	0.995
*stx2 *+ *sta*	1 (0.1)	0 (0)	0.996
*F17 *+ *stx1 *+ *eae*	1 (0.3)	1 (0.3)	0.819
*F5 *+ *stx1 *+ *saa*	1 (0.3)	0 (0)	0.997
Total	178 (54.1)	210 (58.3)	0.420

### Distribution of virulence factors according to age

Potential associations between age and detection of virulence factors were evaluated. As shown in Table 4, the *F17* gene was detected most frequently. The *stx1* and *eae* were detected in all age groups, but their frequency was low, whereas the *saa* gene was found only in calves aged 1–20 days. The *F5* gene was detected in fecal samples from three calves only. The *F41* gene was not observed as a single factor in samples from any of the age groups, although it was detected in combination with *F17* in samples from three calves (Table 4). Except for the *F17*, no statistical significance between any single virulence factors and age was found. Moreover, the combinations of *F17* and *stx1* were detected more frequently with increasing age, a finding that reached critical statistical significance (P = 0.005). Interestingly, there were no interactive effects of age and diarrhea with respect to the prevalence of virulence genes.

**Table 4:** Age distribution of virulence genes detected in fecal samples from pre-weaned calves

Virulence genes	No. (%) of *E. coli* strains by age (days)				Diarrhea × age
	1–20 (n = 337)	21–40 (n = 218)	41–60 (n = 134)	P value	P value
*F17*	104 (30.9)	110 (50.5)	66 (49.3)	< 0.001	0.519
*F5*	2 (0.6)	1 (0.5)	0 (0.0)	1.000	1.000
*sta*	0 (0.0)	1 (0.5)	1 (0.7)	0.161	1.000
*stx1*	18 (5.3)	12 (5.5)	6 (4.5)	0.442	0.290
*stx2*	1 (0.3)	0 (0.0)	0 (0.0)	1.000	1.000
*eae*	10 (3.0)	5 (2.3)	2 (1.5)	0.846	0.158
*saa*	7 (2.1)	0 (0.0)	0 (0.0)	0.186	1.000
*F17 *+ *F5*	0 (0.0)	2 (0.9)	1 (0.7)	0.065	1.000
*F17 *+ *stx1*	3 (0.9)	3 (1.4)	6 (4.5)	0.005	0.858
*F17 *+ *F41*	1 (0.3)	1 (0.5)	1 (0.7)	0.353	1.000
*F17 *+ *eae*	1 (0.3)	1 (0.5)	1 (0.7)	0.353	1.000
*stx1 *+ *eae*	7 (2.1)	6 (2.8)	1 (0.7)	0.258	0.535
*stx1 *+ *saa*	0 (0.0)	1 (0.5)	1 (0.7)	0.161	1.000
*stx1 *+ *F5*	0 (0.0)	1 (0.5)	0 (0.0)	0.401	1.000
*stx2 *+ *sta*	0 (0.0)	1 (0.5)	0 (0.0)	0.401	1.000
*F17 *+ *stx1 *+ *eae*	1 (0.3)	0 (0.0)	1 (0.7)	0.350	1.000
*F5 *+ *stx1 *+ *saa*	1 (0.3)	0 (0.0)	0 (0.0)	1.000	1.000
Total	156 (46.3)	145 (66.5)	87 (64.9)	0.000	0.328

The binomial regression analysis revealed that detection of the *F17* gene was significantly associated with age. The *F17* gene was more prevalent with increasing age: notably, it was detected 2.62-fold more frequently in fecal samples from calves aged 21–40 days (95% CI 1.79–3.84; P < 0.001) and 2.44-fold more frequently in calves aged 41–60 days (95% CI 1.57–3.81; P < 0.001) than in calves aged 1-20 days. The combinations of the *F17* and *stx1* genes were statistically significant in calves aged 41–60 days compared to those aged 1–20 days (OR = 7.70, P = 0.005; Table 5). However, no relationship between diarrhea and the combinations of *F17* and *stx1* genes was observed.

**Table 5:** Age distribution of *Escherichia coli F17* and *F17 *+* stx1* virulence genes detected from pre-weaned Korean native calves

Age (days)	*F17* (positive)			*F17 *+* stx1* (positive)		
	OR	P value	95% CI	OR	P value	95% CI
1–20 (ref.)	1.00	–	–	1.00	–	–
21–40	2.62	< 0.001	1.79–3.84	2.48	0.273	0.49–12.57
41–60	2.44	<0.001	1.57–3.81	7.70	0.005	1.86–31.95

## Discussion

*Escherichia coli* has been reported as among the most common pathogens associated with diarrhea in calves, leading to high morbidity and mortality worldwide. However, remarkably few studies have been performed to evaluate the relationship between *E. coli* and calf diarrhea in the ROK [18–20]. In the present study, we examined the prevalence of *E. coli* according to age and explored the possibility of associations between *E. coli* and diarrhea in pre-weaned calves. Moreover, this study provides important new information regarding *E. coli* as a causative agent and its relationship to the pathogenesis of diarrhea in calves.

With the regard to the relationship between age distribution and prevalence of *E. coli* in fecal samples, our findings indicate that the prevalence of *E. coli* in fecal samples was highest among calves aged 21–40 days (66.5%) and lowest in 1–20-day-old calves (46.3%). *E. coli* is considered to be an important etiological agent of neonatal calf diarrhea, together with rotavirus, coronavirus, and *Cryptosporidium parvum*. However, our findings revealed that the presence of *E. coli* was not directly associated with diarrhea in pre-weaned calves. According to previous studies, *E. coli* can result in diarrhea during the first week of life in calves [1, 3, 11, 21]. However, our findings contradict with these results as the prevalence of *E. coli* was at its lowest in calves under 20 days of age in our study cohort and no mortality due to *E. coli* infection was identified. Interestingly, the incidence of *E. coli* increased significantly in association with the age, most notably after 21 days; we cannot accurately address the reasons underlying this observation, however, this finding also suggests that *E. coli* is unlikely to be a causative agent for diarrhea in this study. As such, we conclude that diarrhea was not caused by *E. coli* itself; however, this condition typically results from complex interactions among various pathotypes and virulence factors of *E. coli* and/or co-infections with other pathogens. While our results cannot fully rule out the possibility that *E. coli* serves as a risk factor for diarrhea in pre-weaned calves, our findings suggest that it is unlikely to be the main causative agent of diarrhea in this study.

ETEC strains are typically regarded as among the most important agents contributing to the pathogenesis of diarrhea. The first step in the infection process is the colonization of the intestinal epithelium, which is mediated by ETEC-associated fimbrial adhesins, primarily F5, F17, and F41 [3]. F5 and F41 have been associated with the incidence of diarrhea among neonatal calves [11]. F5 was the first virulence factor identified in ETEC, while F41 has been detected in both healthy and diarrheic calves. F41 may also have different virotypes; in other words, F41 present in healthy calves might be expressed specifically in response to immune factors associated with stress and diet, thereby promoting the onset of diarrhea. However, in this study, the *F41* gene was detected only in combination with *F17*, and the *F5* gene was found only in three calves. Moreover, the prevalence of F5 may be quite low because farms frequently inoculate calves using a vaccine that targets this antigen.

After their attachment to the intestinal mucosa, ETEC strains produce enterotoxins ST and LT; ST is more prevalent than LT and may play a major role in the pathogenesis of calf diarrhea [11]. The STa enterotoxin is produced by both porcine and bovine ETEC strains, whereas LT and STb enterotoxins are predominantly produced by porcine and human ETEC strains. There is a specific link between ETEC-associated heat-stable toxins and adhesins; STa is significantly linked to the F5/F41 combination [22]. However, the *sta* enterotoxin gene was detected in two calves only; this finding may explain why *F5* and *F41* showed a low prevalence. Taken together, these results suggest that the limited expression of fimbrial adhesins may prevent the ETEC-associated virulence factors from initiating their pathogenic actions by secreting toxins, thereby preventing the onset of diarrhea.

F17 fimbriae have been implicated in the pathogenesis of calf diarrhea as they mediate the adherence of bacterial cells to calf intestinal villi. In this study, *F17* was the most predominant virulence factor detected in the *E. coli* strains isolated from pre-weaned calves in the ROK; *F17* showed at a slightly higher prevalence in normal feces than in diarrheic feces. This could be because F17 may be related to diseases other than diarrhea in cattle. F17 has been detected in human and ruminant necrotoxigenic *E. coli* (NTEC) [23–25]. NTEC is the etiologic agent of extraintestinal infections in humans, pigs, dogs, and cats [26]; it has also been associated with intestinal infections or septicemia in calves [27]. NTEC is detected frequently in healthy calves, although the risk of NTEC infection increases after the first month of life [28]. Our results demonstrated no associations between *F17* detection and diarrhea. According to our findings, the prevalence of *F17* significantly increased after 21 days of age (P < 0.001) compared to those aged 1–10 days. Furthermore, *F17*-positive calves aged 41–60 days were at increased risk of Shiga toxin (Stx1) production (OR = 0.25, 95% CI 0.49–12.57; P = 0.005). However, we were unable to identify any associations between these genes with diarrhea. Another very important point to consider is that these calves may represent potential reservoirs of human pathogenic *E. coli*. The results presented here suggest that *F17* has a stronger association with *E. coli* colonization compared to *F5* and *F41*. Further studies will be needed to determine whether there are any specific correlations between various F17 subtypes and ETEC and NTEC pathotypes.

*Stx1* was identified in 36 (9.3%) of the fecal samples from pre-weaned calves, whereas s*tx2* was identified in one (0.3%) diarrheic calf only. Several studies have reported that Stx1 is observed among groups of diarrheic calves, whereas Stx2 is identified as predominant among those that are healthy [7, 29]. Our results revealed that neither *stx1* nor *stx2* were associated with diarrhea among the calves evaluated in this study. One report suggested that Stx2 may be related to the severity of disease [30]. In humans, infection with isolates producing Stx2 toxins can result in hemorrhagic colitis and HUS [31]. In contrast, our findings suggest that STEC may be associated with fewer problems in cattle than in humans. Among the explanations for this observation, Stx toxins may have no activity at the bovine intestinal epithelium and may be unable to translocate across bovine enterocytes, in contrast to what has been observed in humans. Moreover, in the present study, *stx1* was detected more frequently than *stx2*. Although we could not identify a relationship between diarrhea and Shiga toxin, our results also suggest that calves might act as an important reservoir for STEC infection in humans.

The *eae* gene product promotes interactions between bacteria and epithelial cells in vivo. In this study, the *eae* gene was detected in 2.4% and 2.5% of *E. coli* samples taken from diarrheic and normal feces, respectively. Interestingly, the *eae* gene was not detected in pathogenic *E. coli* strains isolated from cattle feces and its low prevalence has been reported in many studies [32, 33]. The role of *eae* in promoting the pathogenesis of diarrhea remains unclear. Of note, the *saa* was previously associated with an outbreak of HUS [10]. In addition, the *saa* gene was detected in seven calves (1.8%). These results support our observations regarding the virulence factors of STEC strains and the fact that they are unlikely to be involved in pathogenicity in the gastrointestinal tracts of calves. Consequently, the present study also highlights the important observation that healthy calves may serve as a potential reservoir for STEC.

## Conclusions

The present results indicate that *F17* is predominant among the virulence genes detected in fecal samples from pre-weaned calves in the ROK. Our findings revealed no associations between diarrhea and detection of ETEC or STEC strains. The incidence of *E. coli* was found to be age-related, although the detection of *E. coli* was not associated with diarrhea. Taken together, these results suggest that *E. coli* may serve as a risk factor and reservoir for STEC infection in humans rather than as a causative agent of calf diarrhea. This study provides useful information on the virulence factors characteristic of *E. coli* that are prevalent in the ROK. As such, these findings constitute an important contribution to our understanding of gastrointestinal disorders in calves and likewise provide an important database for the implementation of diagnostic, preventive, and treatment measures.

## Data Availability

All data generated or analyzed during this study are included in the article.
